# Genetic Inactivation of the Serotonin Transporter Dysregulates Expression of Neurotransmission Genes and Genome‐Wide DNA Methylation Levels in the Medial Prefrontal Cortex of Male Rats During Postnatal Development

**DOI:** 10.1002/dneu.22973

**Published:** 2025-06-08

**Authors:** Yvet Kroeze, Martin Oti, Roel H. M. Cooijmans, Ellen van Beusekom, Leonie I. Kroeze, Anthonieke Middelman, Hans van Bokhoven, Sharon M. Kolk, Judith R. Homberg, Huiqing Zhou

**Affiliations:** ^1^ Donders Institute for Brain, Cognition, and Behaviour, Centre for Neuroscience, Department of Medical Neuroscience Radboud University Medical Center Nijmegen Gelderland the Netherlands; ^2^ Department of Human Genetics Radboud University Medical Center, Donders Institute for Brain, Cognition, and Behaviour, Centre for Neuroscience Nijmegen Gelderland the Netherlands; ^3^ Department of Molecular Developmental Biology Faculty of Science Radboud University, Radboud Institute for Molecular Life Sciences Nijmegen Gelderland the Netherlands; ^4^ Department of Laboratory Medicine Laboratory of Hematology Radboud University Medical Center Nijmegen Gelderland the Netherlands; ^5^ Donders Institute for Brain, Cognition, and Behaviour, Centre for Neuroscience, Department of Molecular Animal Physiology Radboud University Nijmegen Gelderland the Netherlands

**Keywords:** development, prefrontal cortex, rat, RNA‐seq, serotonin transporter

## Abstract

Reduced expression of the serotonin transporter (5‐hydroxytryptamine transporter, 5‐HTT) in early life has been associated with a delay in postnatal brain development and endophenotypes of a variety of neuropsychiatric and neurodevelopmental disorders in adolescence and adulthood. How a reduction in functional 5‐HTT can disrupt neurodevelopment is still largely unknown. Here, we studied genome‐wide gene expression using transcriptome analysis (RNA‐seq) and global levels of DNA (hydroxy)methylation (5(h)mC) using high‐performance liquid chromatography‐tandem mass spectrometry in 5‐HTT wild‐type (5‐HTT^+/+^) and 5‐HTT homozygous knockout (5‐HTT^−/−^) rats across life (postnatal day [PND] 8, 14, 21, 35, and 70) in the medial prefrontal cortex (mPFC); a brain region with an extensive serotonergic innervation involved in several neuropsychiatric endophenotypes. We observed most gene expression changes in the mPFC during early postnatal life (PND8) and found at this time point an enrichment of genes linked to neuronal and developmental processes like neurotransmission, neuropeptide signaling, and cell migration. Genome‐wide DNA 5(h)mC analysis showed a global increase in 5‐hydroxymethylcytosine (5hmC) in the mPFC during development in both genotypes and a significant increase in global 5hmC in 5‐HTT^−/−^ compared to 5‐HTT^+/+^ rats at PND35. The differences in the regulation of gene expression in 5‐HTT^−/−^ versus 5‐HTT^+/+^ rats during early postnatal life can dysregulate neurodevelopmental processes resulting in aberrant brain wiring and functioning. This can result in lifelong consequences for prefrontal context‐dependent executive functioning.

## 1 Introduction

Serotonin (5‐hydroxytryptamine; 5‐HT) is an important neurotransmitter in the central nervous system, where it has various functions, including the regulation of mood (Young and Leyton 2002), appetite (Tecott 2007), sleep (Silber and Schmitt 2010; Ivarsson et al. 2005), and learning and memory (Schmitt et al. 2000; Buhot 1997). 5‐HT is produced by serotonergic neurons in the raphe nuclei, which send their axons to regions throughout the brain. The serotonin transporter (5‐hydroxytryptamine transporter [5‐HTT], encoded by *SLC6A4*) facilitates the reuptake of extracellular 5‐HT in presynaptic serotonergic neurons and thereby regulates extracellular 5‐HT levels available for the activation of 5‐HT receptors located at pre‐ and postsynaptic neurons. Besides its role as a neurotransmitter, 5‐HT also acts as an important trophic factor during neurodevelopment. 5‐HT can promote neurodevelopmental processes, such as migration, neuronal outgrowth, and synaptogenesis (Faber and Haring 1999; Witteveen et al. 2013; Gaspar et al. 2003; Riccio et al. 2009; Fricker et al. 2005). In addition, although in the adult brain 5‐HTT is only expressed in raphe neurons, 5‐HTT expression is more widespread at early developmental stages. At mid‐gestation (embryonic day 10.5 in rodents; Gaspar et al. 2003), expression of 5‐HTT begins in the 5‐HT neurons of the raphe nuclei, and subsequently 5‐HTT expression emerges in the non‐serotonergic (glutamatergic) neurons of the sensory system and corticolimbic pathway, including the prefrontal cortex (Narboux‐Neme et al. 2008; Homberg et al. 2010). These neurons are termed serotonin‐absorbing neurons and serve to prevent excessive levels of serotonin in the extracellular space (Gaspar et al. 2003; D'Amato et al. 1987). During the second postnatal week, the 5‐HTT expression in non‐serotonergic neurons ends rapidly (Homberg et al. 2010), and from then 5‐HTT is only expressed in the serotonergic neurons from the raphe nucleus.

The medial prefrontal cortex (mPFC), a brain region involved in the execution of cognitive and emotional functions (Kolb et al. 2012; Euston et al. 2012; Feja and Koch 2014; Siddiqui et al. 2008; Amodio and Frith 2006), has an extensive serotonergic innervation (Wilson and Molliver 1991; Hoover and Vertes 2007) and has been implicated in neuropsychiatric disorders like anxiety, depression, and drug addiction (Vialou et al. 2014; Albert et al. 2014; Goldstein and Volkow 2011). The mPFC is one of the latest brain regions to mature, showing substantial changes during postnatal development (Fuster 2002; Fung et al. 2010). In explant cultures from developing 5‐HTT knockout (5‐HTT^−/−^) rat brains (embryonic day 16.5), 5‐HT projections from the median raphe are strongly attracted by the mPFC, suggesting that 5‐HT levels can influence connectivity in the mPFC (Witteveen et al. 2013). Indeed, the layer identity of the mPFC neurons and reelin‐positive interneuron number and integration are altered in the embryonic and early postnatal brain in the absence of the 5‐HTT (Garcia et al. 2019; Brivio et al. 2019). In addition, the number of a class of callosal projection neurons is decreased in the developing 5‐HTT^−/−^ mPFC compared to 5‐HTT^+/+^ mPFC (Witteveen et al. 2013). It has furthermore been reported that the expression of proteins essential for the glutamatergic synapses is reduced in the PFC of 5‐HTT^−/−^ rats across development, from preweaning to juvenile and adult stages (Brivio et al. 2019). Moreover, brain‐derived neurotrophic factor (BDNF) mRNA and protein expression was found to be reduced at birth in the mPFC of 5‐HTT^−/−^ versus 5‐HTT^+/+^ rats. The magnitude of these changes became more pronounced starting from weaning, being sustained by epigenetic mechanisms as well as alterations in the expression of specific transcription factors (Calabrese et al. 2013). Adult 5‐HTT^−/−^ mice did not show changes in BDNF in the prefrontal cortex as measured by ELISA (Kronenberg et al. 2016). 5‐HTT^−/−^ mice do show abnormally distributed interneurons and altered neocortical cell density and layer thickness (Altamura et al. 2007), caused by disrupted migration of neurons during development (Riccio et al. 2009). Furthermore, in adult 5‐HTT^−/−^ mice, the apical dendritic branches of infralimbic cortical pyramidal neurons were significantly increased in length (Wellman et al. 2007). Finally, 1 week old 5‐HTT^−/−^ mice show changes in the expression of genes involved in cytoskeleton interactions, neurite/axon outgrowth, and synapse development in the prefrontal cortex (Soiza‐Reilly et al. 2019). In humans, the low‐activity short (S) allelic variant of the serotonin transporter promoter‐linked polymorphic region (5‐HTTLPR), which is associated with reduced transcription of the 5‐HTT gene (Heils et al. 1996), has been shown to reduce mPFC activity (Schipper et al. 2019). Furthermore, children and adolescents carrying a homozygous S‐allele (S/S) showed weaker connectivity in the superior mPFC compared to l‐allele carriers (Wiggins et al. 2012). Taken together, the absence of the 5‐HTT has profound effects on the development of the mPFC.

Given the central role of the PFC in cognitive processes, the 5‐HT‐dependent changes in PFC development and function can have profound consequences. As such, mPFC‐regulated behavior is altered in 5‐HTT^−/−^ rats. More specifically, these animals display increased anxiety (Kalueff et al. 2010; Olivier et al. 2008), depression‐like behavior (Olivier et al. 2008; Wellman et al. 2007; Sbrini et al. 2020), behavioral and cognitive flexibility (Nonkes et al. 2013), reduced impulsivity (Homberg et al. 2007), reduced aggression (Homberg et al. 2007), a fear extinction deficit (Schipper et al. 2019), and compulsive cocaine self‐administration behavior at adulthood (Muller and Homberg 2015). During preadolescence, the animals display reduced social play (Homberg et al. 2007) and a fear extinction deficit (Schipper et al. 2019). In humans, the 5‐HTTLPR s‐allele is associated with differences in mPFC‐related behavior, like sustained attention (Roiser et al. 2007), cognitive flexibility (Weiss et al. 2014), and decision making (Crisan et al. 2009), as well as increased risk for developing depression (Caspi et al. 2003), anxiety (Lesch et al. 1996), and substance abuse (Muller and Homberg 2015). These rodent and human data together imply that 5‐HTT downregulation in early life may contribute to vulnerability to the development of major neuropsychiatric disorders.

Neurodevelopment is a highly complex and strictly regulated process that is dependent on the precisely timed and coordinated expression of thousands of genes. Each specific neurodevelopmental process requires a different set of genes to be expressed at a specific time frame during development. There are several studies that investigated the influence of dysregulated 5‐HT signaling on gene expression, showing altered expression of genes linked to the inflammatory system, the hypothalamus–pituitary–adrenal axis, neurotrophic factors, myelination, and neurotransmitter systems. However, most of these studies focused on the effect in adulthood (Kroeze et al. 2012). Studies on 5‐HTT‐dependent gene expression changes across neurodevelopmental stages are limited. Studying genome‐wide gene expression in the mPFC during development might reveal important new insights in the molecular underpinnings of the behavioral changes observed in those characterized by inherited 5‐HTT downregulation.

DNA methylation, an epigenetic mechanism, has also been shown to play an important role in neurodevelopment and in neuropsychiatric disorders (Szulwach et al. 2011). DNA methylation of cytosines at CpG sequences is catalyzed by a family of DNA methyltransferases (DNMTs), which show a spatiotemporal distribution during neurodevelopment. 5‐Hydroxymethylcytosine (5hmC), created by oxidation of 5‐methylcytosine (5mC) by TET proteins, is thought to be an intermediate step in the active demethylation process (Kroeze et al. 2014). However, more recent studies have indicated that 5hmC does not only serve as a DNA demethylation intermediate but also functions as a stable epigenetic mark (Dawlaty et al. 2014; Kafer et al. 2016). Dysregulation of DNA methylation is seen in 5‐HT‐related neurodevelopmental and neuropsychiatric disorders, like autism spectrum disorder (Nardone et al. 2014), schizophrenia (Numata et al. 2014), bipolar disorder (Fries et al. 2016), anxiety (Murphy et al. 2015), and depression (Cordova‐Palomera et al. 2015). As to whether the DNA methylation changes are a cause or consequence of these diseases and whether 5‐HT dysregulation is involved in the methylation changes is unknown. Genome‐wide gene expression and DNA (hydroxy)methylation changes during neurodevelopment because of reduced functional 5‐HTT has been unexplored and may further enrich our understanding of the 5‐HTT‐dependent molecular changes during mPFC development.

To unravel the molecular mechanisms contributing to the structural and behavioral changes in the mPFC related to inherited 5‐HTT downregulation, we studied genome‐wide gene expression using transcriptome analysis (RNA‐sequencing, RNA‐seq) in the mPFC of 5‐HTT^+/+^ and 5‐HTT^−/−^ rats sacrificed at postnatal day (PND) 8, 14, 21, 35, and 70, ages that correspond to the neonatal, “toddler,” juvenile, early adolescent, and adult stages at which most critical developmental processes in the mPFC occur (Chini and Hanganu‐Opatz 2021). We performed pair‐wise comparisons and observed the largest number of differentially expressed genes during early postnatal life (PND8), with an enrichment for genes involved in neurotransmission and cell migration. In addition, we measured genome‐wide DNA (hydroxy)methylation in mPFC tissue from 5‐HTT^+/+^ and 5‐HTT^−/−^ rats sacrificed at PND8, 14, 21, 35, and 70. We observed a significant increase in 5hmC levels in the 5‐HTT^−/−^ rats compared to the 5‐HTT^+/+^ rats at PND35.

## 2 Materials and Methods

### 2.1 Animals

5‐HTT^−/−^ rats (*Slc6a41Hubr*) were generated by ENU‐induced mutagenesis (Smits et al. 2006). Rats were housed in pairs in individually ventilated cages (40 × 35 × 23 cm^3^, Greenline, Tecniplast, West Chester, USA) in temperature‐controlled rooms (21°C ± 1°C) under standard 12‐h light/dark cycle (lights on at 7:00 a.m.) with food (Sniff, long cut pellet, Bio Services, Uden, the Netherlands) and water available ad libitum. Male pups were weaned at PND22. Rats were sacrificed by decapitation at five different time points: PND8, PND14, PND21, PND35, and PND70. For Group 1, 10 wild‐type (5‐HTT^+/+^) and ten 5‐HTT^−/−^ rats were sacrificed from three different nests per genotype per time point and were used for genome‐wide expression analysis and quantitative reverse transcription PCR (RT‐qPCR) experiments. Half of the rats (*n* = 5 rats/genotype/time point) were used for RNA‐seq and RT‐qPCR and the other half (*n* = 5 rats/genotype/time point) only for RT‐qPCR validation. For Group 2, five rats were sacrificed per genotype per time point and used for genome‐wide DNA (hydroxy)methylation analysis. Group 3 was used for validation of the results from Group 2 and consisted of ten 5‐HTT^−/−^ and ten 5‐HTT^+/+^ rats sacrificed at PND35.

### 2.2 RNA Extraction and Double‐Stranded Complementary DNA (cDNA) Synthesis

Five samples per genotype per time point were used for RNA extraction. mPFC tissue was dissected from seven (PND8 and 14) or eight (PND21, 35, and 70) consecutive slices of 200 µm using a 2 mm punch needle and included the prelimbic and infralimbic cortex. For more details about the punching, see Kroeze et al. (2017). Total RNA was isolated with QIAzol (RNeasy lipid tissue kit; QIAGEN, Venlo, the Netherlands) according to the manufacturer's recommendations. From each sample, 2.5 µg RNA was used for rRNA depletion using the Ribo‐Zero rRNA Removal Kit (Human/Mouse/Rat, Epicentre, Madison, Wisconsin, USA) according to the manufacturer's recommendations. RNA fragmentation reactions were performed using fragmentation buffer (5×; 200 mM Tris–acetate, 500 mM potassium‐acetate, 150 mM magnesium‐acetate, pH 8.2) in a final concentration of 1× per reaction. Fragmentation reactions were incubated at 95°C for 4 min on a thermal cycler and placed on ice for 10 min. Fragmented rRNA‐depleted RNA was purified using ethanol precipitation. First and second strand synthesis was performed as described by Kouwenhoven et al. (2015).

### 2.3 Sequencing

DNA samples were prepared for sequencing by end repair of 5 ng total DNA as measured by Qubit dsDNA HS (Invitrogen). NEXTflex adaptors (Bioo Scientific, Austin, Texas, USA) were ligated to the DNA fragments, followed by post‐ligation clean‐up using Agencourt AMPure XP beads (Beckman Coulter, Woerden, the Netherlands), library amplification by PCR (10 cycles), and size selection (∼300 bp) using Agencourt AMPure XP beads (Beckman Coulter). Quality control of DNA libraries prepared for sequencing was performed by qPCR and by running the products on a Bioanalyzer (Bio‐Rad, Veenendaal, the Netherlands). Cluster generation and sequencing (50 bp, single‐end reads) were performed with the Illumina HiSeq 2000 sequencer according to standard Illumina protocols. Samples were sequenced to a depth of approximately 29 million reads per sample.

### 2.4 RNA‐Seq Data Processing

Reads were aligned to the rn4 rat genome assembly using the GSNAP program (Wu and Nacu 2010), version 2012‐07‐20. Transcript quantification analysis was done with Cufflinks (Trapnell et al. 2010) using the rat Ensembl transcriptome (version 69). Cufflinks quantify gene expression as FPKM (fragments per kilobase per million mapped reads), which corrects for different transcript lengths and sample sequencing depths. Short genes (5201 genes shorter than 200 base pairs, including small nuclear RNAs, small nucleolar RNAs, microRNAs, and miscellaneous RNAs) were removed. This is because FPKMs of short genes tend to be more affected by background noise, as the correction for gene length gives more weight to the reads on short genes, thereby possibly increasing the FPKM to biologically irrelevant levels. To check for possible mistakes and errors during the sequencing of the RNA, the reads were assessed for the quality of calling the nucleotides in the reads quantified by Phred scores and the enrichment of not‐called bases at specific locations in the reads (Andrews 2010). The FPKMs calculated by Cufflinks were used to perform a principal component analysis (PCA) to look at variance between the samples (R‐Foundation 2015). PCA was performed on log10‐transformed FPKM values.

### 2.5 Differential Expression Analysis, Clustering, and Gene Function

Differential expression analysis was performed using the CuffDiff program (Trapnell et al. 2013) version 2.2.1, using the Ensembl database (Flicek et al. 2014) version 69 rat transcriptome annotation. The expression values from Cufflinks were further normalized by CuffDiff across the samples in the experiment by scaling them according to the geometric mean of the samples. On the basis of these expression data, CuffDiff calculates differentially expressed genes (*p* < 0.01, FPKM > 1, fold change > 1.2) between the 5‐HTT^−/−^ and 5‐HTT^+/+^ rats for all five time points, taking replicates per genotype into account. For the hierarchical clustering, the mean FPKM across replicates was taken per condition for all genes, and the coefficient of variation (CV; standard deviation/mean) across all conditions was calculated. This was used to filter for highly variable genes with a CV > 0.3 (3662 genes). This threshold was chosen on the basis of the plotting the distribution of CVs across all genes (Figure S1) and visually identifying the approximate start of the right tail of the distribution. Subsequently, the FPKMs were standardized (*Z*‐scores) and plotted with the R pheatmap package (Kolde 2019), using the Euclidean distance metric and average linkage. The samples were manually ordered according to the PND in visualization, without changing the cluster tree structure. Several genes were manually labeled into the plot, according to their gene expression patterns. Gene ontology (GO) analysis was performed using the DAVID website (Huang da et al. 2009). DAVID calculates modified Fisher Exact *p* values, EASE scores, for each gene category (Huang et al. 2009). Ingenuity Pathway Analysis software package (Ingenuity Systems, www.ingenuity.com) was used for pathway analysis and analysis of top diseases and bio functions. Ingenuity calculates *p* values for the enrichment of each gene category using the right‐tailed Fisher exact test, taking into consideration both the total number of molecules from the analyzed dataset and the total number of molecules linked to the same gene category according to the Ingenuity Knowledge Base. We generated a molecular network by integrating the results of the bioinformatics analyses from PND8 with systematic literature searches. For all genes linked to one of the five most significantly enriched GO‐terms and genes linked to one of the five most significantly enriched ingenuity pathways (except hepatic fibrosis/hepatic stellate cell activation), we looked at the available information in the NCBI Gene database and subsequently searched PubMed using the search terms “prefrontal cortex,” “neurodevelopment,” “signal transduction,” “serotonin,” “glutamate,” in combination with the name of each candidate gene or their protein name. Guided by the literature we found, we also searched PubMed for functional interactions between the candidate genes/encoded proteins.

### 2.6 Quantitative Reverse Transcription PCR

RNA‐seq validation was performed by RT‐qPCR analysis of 10 selected genes. Primers were designed using Primer3 online software (http://frodo.wi.mit.edu). See Table S1 for primer sequences. cDNA was synthesized using 500 ng of total RNA in a reverse transcription reaction using iScript cDNA Synthesis Kit according to manufacturer's protocol (Bio‐Rad, Veenendaal, the Netherlands). qPCR reactions were performed in a 7500 Fast Real Time PCR System (Applied Biosystems, Foster City, CA, USA) using the SYBR Green fluorescence quantification system (GoTaq qPCR Master Mix, Promega Benelux BV, Leiden, the Netherlands). Thermal cycling was initiated with incubation at 95°C for 10 min followed by 40 cycles of 95°C for 30 s and 60°C for 1 min. To normalize the cDNA content of the samples, we used the comparative threshold cycle (CT) method (Livak and Schmittgen 2001), which consists of the normalization of the number of target gene copies versus an endogenous reference gene *Gabbr1* (stable gene in the RNA‐seq dataset throughout all ages based on the CV (CV = stddev/mean).

### 2.7 Genome‐Wide DNA (Hydroxy)Methylation

DNA of mPFC tissue was isolated using the ReliaPrep gDNA Tissue miniprep system (Promega, Leiden, the Netherlands) according to the manufacturer's recommendations. An amount of 200 ng of DNA was degraded into individual nucleosides using DNA degradase plus (Zymo Research, Irvine, CA). 5mC and 5hmC levels were measured as described by Kroeze et al. (2014). In short, the individual nucleosides (dG, mdC, and hmdC) were measured using a high‐performance liquid chromatography–tandem mass spectrometry (HPLC–MS/MS) system (Waters, Milford, MA, USA). Calibration standards containing internal standard solutions were measured to obtain area‐based linear regression curves for quantification. The 5mC and 5hmC levels were calculated as a concentration percentage ratio of % 5‐methyl‐2′‐deoxycytidine/2′‐deoxyguanosine (%mdC/dG) and % 5‐hydroxymethyl‐2′‐deoxycytidine/2′‐deoxyguanosine (%hmdC/dG), respectively.

### 2.8 Statistical Analysis

Statistical analysis of RT‐qPCR and 5(h)mC data was carried out using the IBM Corporation Statistical Package for the Social Sciences (SPSS) version 20.0 (IBM Corp., Armonk, NY, USA). Data were analyzed using independent samples *t‐*tests (corrected *p* value was used when equal variance was not assumed). Outliers (data points further than three interquartile ranges from the nearer edge of the box plot) were excluded from the analysis. *t‐*tests were performed two‐sided, and the level of statistical significance was set at *p* < 0.05.

## 3 Results

### 3.1 Validation of the Knockout Model and General Differences Between PFC Samples

RNA‐seq was performed, and the reads were aligned to the rn4 rat genome assembly. To validate our knockout model, reads spanning the third exon of the *Slc6a4* gene, corresponding to chromosome 10 position 67152790 to 67153009 from the Brown Norway rat genome (rn4 assembly; Consortium RGSP 2004), were extracted and examined for the described mutation by calling differences between the extracted reads and the reference genome (Li et al. 2009). In the 5‐HTT^−/−^ rats, this third exon should contain a C to A transversion at nucleotide 3924 (c.3924C > A, based on ENSRNOG0000003476), resulting in a premature stop codon (p.C1308X) (Homberg et al. 2007). All 5‐HTT^+/+^ and 5‐HTT^−/−^ replicates were pooled because the coverage of the 5‐HTT gene by individual samples was too low. The third exon was more extensively covered in the 5‐HTT^+/+^ samples (23 reads) compared to the 5‐HTT^−/−^ samples (9 reads). Indeed, the 5‐HTT^−/−^ rat differed from the 5‐HTT^+/+^ rat the reference at nucleotide position 3924, matching the described mutation by Homberg et al. (2007). It should be noted that this variant was based on only a single read that covers this genomic region (Figure S2A). In addition, the 5‐HTT^+/+^ and 5‐HTT^−/−^ sequences both differed from the reference at nucleotide position 3891, indicating a single nucleotide polymorphism (rs8154473, encoding for the same amino acid) between the reference (Brown Norway rat) and our Wistar rats (Figure S2A, deviations highlighted in orange).

Differential expression analysis with CuffDiff (Trapnell et al. 2010) was performed to obtain the normalized gene expression values (FPKM values). A PCA was performed on log10‐transformed FPKM values to examine variations between the samples. Principal component 1 (PC1) (19.2%) seems to separate samples according to age, with the PND8 samples on the left side and the PND70 samples on the right side (Figure S2B). PC2 (6.7%) separated four samples from the rest because these four samples were from different time points (PND8, 14, 21, 35) and from both 5‐HTT^+/+^ and 5‐HTT^−/−^ rats, indicating that these might be potential outliers. Therefore, we decided to exclude them for further analyses. Samples of 5‐HTT^+/+^ and 5‐HTT^−/−^ rats are not clearly separated in this analysis, indicating small gene expression differences in mPFCs between 5‐HTT^+/+^ and 5‐HTT^−/−^ rats.

### 3.2 Differential Expression Analysis and Clustering

**FIGURE 1:**
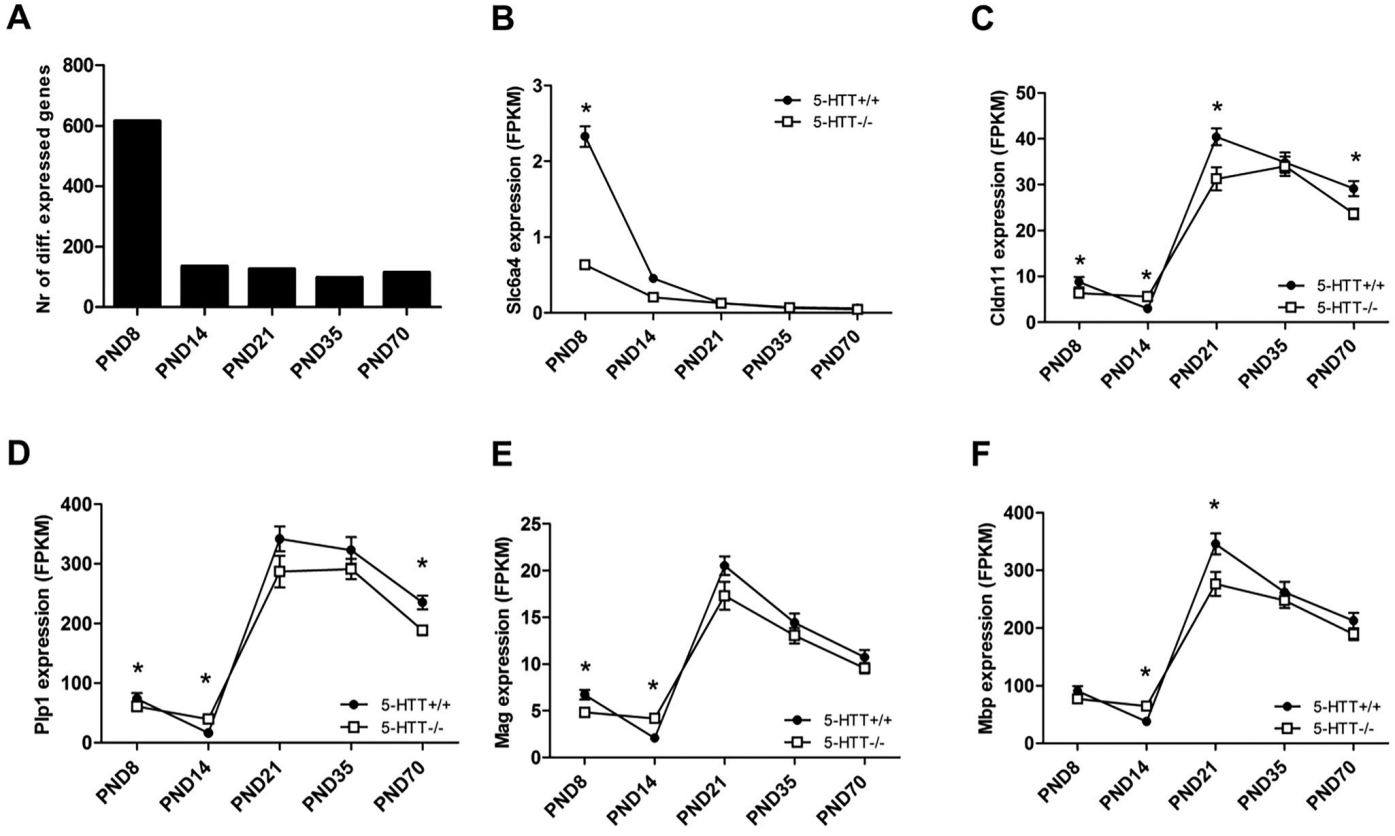
Differential expression analysis in prefrontal cortex RNA of 5‐HTT^−/−^ and 5‐HTT^+/+^ rats. (A) The number of differentially expressed genes per time point. (B–F) Graphs of expression patterns of the serotonin transporter (5‐HTT) gene and four myelin‐related genes. Fragments per kilobase per million mapped reads (FPKM) at five developmental time points in 5‐HTT^−/−^ and 5‐HTT^+/+^ medial prefrontal cortex are shown. (B) *Slc6a4*, (C) *Cldn11*, (D) *Plp1*, (E) *Mag*, (F) *Mbp*. *p < 0.01 based on RNA‐seq statistical analysis.

For each time point, differentially expressed genes (*p* < 0.01, FPKM > 1, fold change > 1.2) between 5‐HTT^−/−^ and 5‐HTT^+/+^ rats were determined using four to five samples per genotype. The highest number of differentially expressed genes between 5‐HTT^−/−^ and 5‐HTT^+/+^ rats was found at PND8 (615 genes). The other time points showed lower numbers of differentially expressed genes, namely, 135 genes for PND14, 126 genes for PND21, 97 genes for PND35, and 115 genes for PND70 (Figure 1A, Table S2). The *Slc6a4* gene (encoding for 5‐HTT) showed differential expression only at PND8 (Figure 1B), and its expression at the other time points was low. Consequently, no significant differences were observed at these time points. Of the differentially expressed genes, 123 genes were differentially expressed at more than one time point. None of these genes showed differential expression at all five time points. Manual checking for these 123 differentially regulated genes with overlapping function revealed four genes involved in myelination (*Cldn11, Plp1, Mag, Mbp* (Kristiansen et al. 2009)), which is a process that has been associated with 5‐HT signaling before (Simpson et al. 2011; Kroeze et al. 2015; Fan et al. 2014). All four genes showed differential expression (*p* < 0.01) between 5‐HTT^−/−^ and 5‐HTT^+/+^ rats at more than one time point, and all showed a similar expression pattern (Figure 1C–F). Although the expression difference of these genes between 5‐HTT^−/−^ and 5‐HTT^+/+^ rats did not reach significance (*p* < 0.01) at all time points, in general, we observed a lower expression of myelin‐related genes in 5‐HTT^−/−^ rats compared to the 5‐HTT^+/+^ rats at PND8, 21, and 70 and a higher expression in 5‐HTT^−/−^ compared to 5‐HTT^+/+^ rats at PND14 (Figure 1C–F).

**FIGURE 2:**
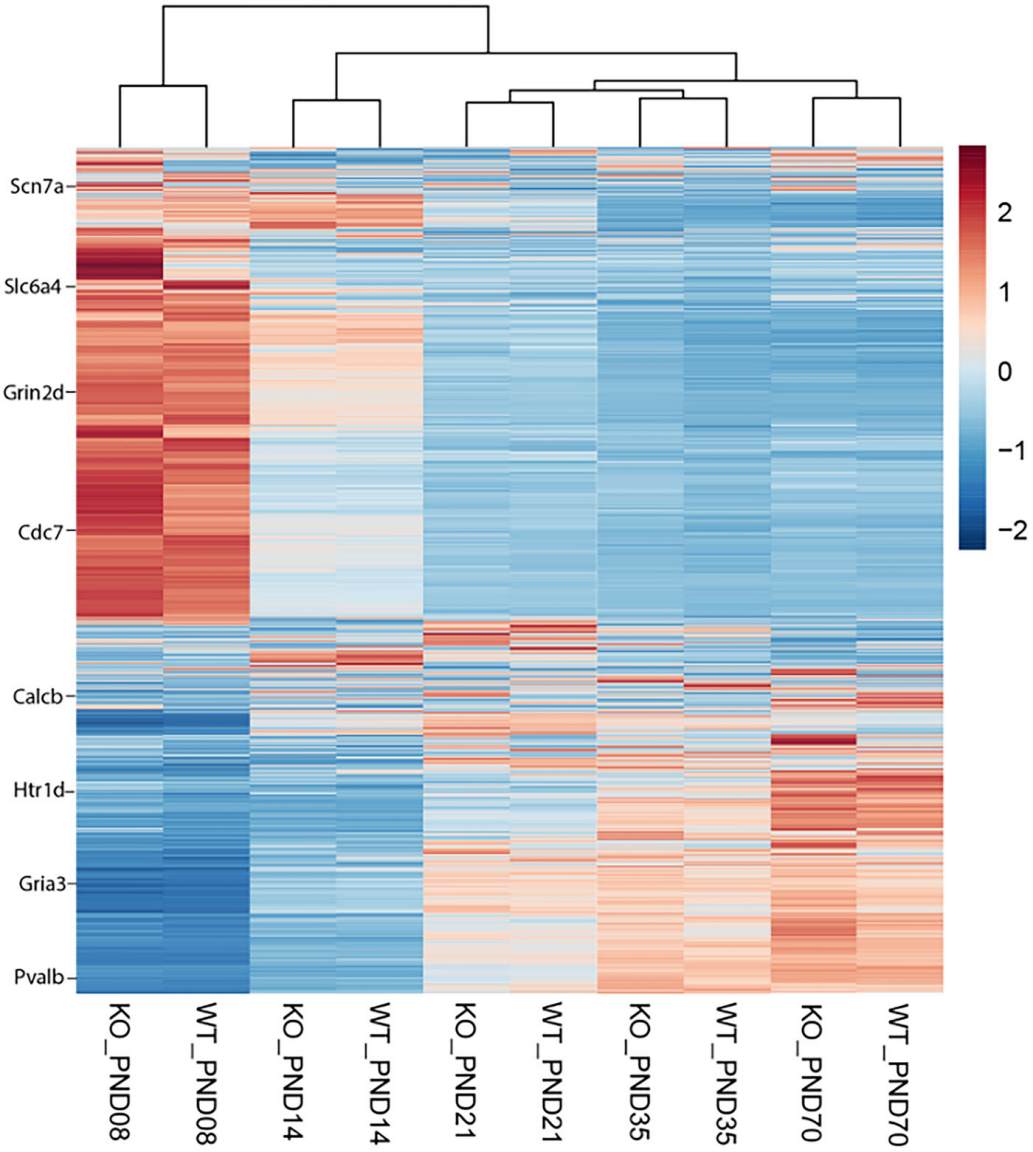
Hierarchical clustering of the differentially expressed genes in 5‐HTT^−/−^ and 5‐HTT^+/+^ rats. Hierarchical clustering of the highly variable genes between 5‐HTT^+/+^ and 5‐HTT^−/−^ samples. The genes are filtered for a coefficient of variation >0.3, standardized per gene (*Z*‐scores) and clustered with the Euclidean distance metric and average linkage algorithm. Red indicates above average expression and blue indicates below average expression. Example genes from the excel file (Table S6) underlying this dataset are shown.

The highly variable genes (all time points combined) were clustered with hierarchical clustering based on *Z*‐scores (Figure 2, Supporting Information Excel File 1). The 5‐HTT^+/+^ and 5‐HTT^−/−^ cluster together per age, indicating that in the group of differentially expressed genes between the 5‐HTT^+/+^ and 5‐HTT^−/−^ mPFC samples, the differences in time were larger than the differences between genotypes. In addition, gene expression patterns seem to differ most between early (PND8 and 14) and later ages (PND21, 35, and 70). These results are consistent with the data from the PCA (Figure S2).

In conclusion, most differences in gene expression in the mPFC were observed at the earliest time point, PND8. These expression differences might affect developmental processes which can result in lifelong consequences for brain functioning.

### 3.3 Gene Ontology and Pathway Analysis

To functionally categorize the differentially expressed genes, GO analysis was performed. The most significantly enriched GO‐terms for the different time points were **transmission of nerve impulse** (PND8), **gas transport** (PND14), **response to organic substance** (PND21), **translational elongation** (PND35), and **response to nicotine** (PND70). See Table 1 for the top five GO‐terms per time point. For a complete list of GO‐terms, see the Supporting Information table. In addition, we used ingenuity which is a tool that models, analyzes, and understands complex biological systems by linking incoming data with known information regarding molecular interactions, cellular phenotypes, and disease processes. Ingenuity core analysis was used to look for significantly enriched pathways. The most significantly enriched pathways for the different time points were **G‐protein‐coupled receptor signaling** (PND8), **neuroprotective role of THOP1 in Alzheimer's disease** (PND14), **chondroitin sulfate degradation** (Metazoa) (PND21), **EIF2 signaling** (PND35), and **growth hormone signaling** (PND70) (Table 2). As at PND14, 21, 35, and 70, a relative low number of differentially expressed genes (*n* < 135) were found, and the pathway analysis showed higher *p* values and was therefore less reliable for these time points compared to PND8.

**TABLE 1:** Significantly enriched gene onthology (GO)‐terms (biological process) affected by 5‐HTT^−/−^ in the medial prefrontal cortex.

	GO‐term	Genes (nr and examples)	*p* value
**PND8**	Transmission of nerve impulse	29 (e.g., *Slc6a4, Svb2, Vamp2, Grm2*)	3,63E − 08
	Neuropeptide signaling pathway	14 (e.g., *Cartpt, Nmur2, Sstr2, Pdyn*)	1,77E − 07
	Synaptic transmission	24 (e.g., *Slc6a4, Svb2, Vamp2, Grm2*)	2,13E − 07
	Cell–cell signaling	30 (e.g., *Slc6a4, Svb2, Vamp2, Grm2*)	2,70E − 07
	Cell migration	26 (e.g., *Gja1, Smo, Vamp2, Met*)	3,21E − 07
**PND14**	Gas transport	5 (e.g., *Hba‐a2, Hbb, Aqp1, Loc689064*)	6,16E − 06
	Oxygen transport	4 (e.g., *Hba‐a2, Hbb, Loc360504, Loc689064*)	6,80E − 05
	Regulation of acute inflammatory response	4 (e.g., *Npy5r, Anxa1, Cd46, Cr1l*)	1,26E − 03
	Cell adhesion	11 (e.g., *Cntnap5b, Mag, Cldn11, Ctgf*)	1,34E − 03
	Biological adhesion	11 (e.g., *Cntnap5b, Mag, Cldn11, Ctgf*)	1,34E − 03
**PND21**	Response to organic substance	18 (e.g., *P2rx4, Gng7, Igf2, Fos*)	3,37E − 05
	Chemical homeostasis	13 (e.g., *Cartpt, Vgf, Egr2, Cldn11*)	3,96E − 05
	Regulation of action potential in neuron	6 (e.g., *Cldn11 Mbp, Pllp, Mal*)	5,38E − 05
	Response to extracellular stimulus	10 (e.g., *Cartpt, Igf2, Fos, A2m*)	8,92E − 05
	Homeostatic process	15 (e.g., *Cartpt, Vgf, Egr2, Cldn11*)	9,20E − 05
**PND35**	Translational elongation	5 (e.g., *Rpl10, Rpl19, Rps16, Rps7*)	1,03E − 03
	Regulation of blood pressure	4 (e.g., *Cyp11b1, Ptgs2, Calca, Ephx2*)	1,36E − 02
	Translation	7 (e.g., *Rpl27a, rpl10, Rpl19, Rps16*)	1,50E − 02
	Response to glucocorticoid stimulus	4 (e.g., *Fos, Ptgs2, Dusp1, Plat*)	2,06E − 02
	Response to corticosteroid stimulus	4 (e.g., *Fos, Ptgs2, Dusp1, Plat*)	2,38E − 02
**PND70**	Response to nicotine	3 (e.g., *Igf2, Htr2c, Chrna5*)	1,01E − 02
	Fatty acid metabolic process	5 (e.g., *Crem, Acsm3, Plp1, Decr1*)	1,93E − 02
	Cell adhesion	7 (e.g., *Pcdhga1, Hapln4, Nlgn2, Cldn11*)	4,01E − 02
	Biological adhesion	7 (e.g., *Pcdhga1, Hapln4, Nlgn2, Cldn11*)	4,01E − 02
	Behavioral response to nicotine	2 (e.g., *Htr2c, Chrna5*)	4,29E − 02

**TABLE 2:** Top canonical pathways affected by 5‐HTT^−/−^ in the medial prefrontal cortex.

	Pathway	*p* value	Overlap	Example genes
**PND8**	G‐protein‐coupled receptor signaling	2.76E − 06	8.0% 20/251	*Grm2, Grm4*, *Htr4, Htr2a, Rgs4*
	Hepatic fibrosis/Hepatic stellate cell activation	8.52E − 06	8.7% 16/183	*A2m, Col19a1, Col1a2, Igf2, Igfbp3*
	cAMP‐mediated signaling	1.69E − 05	7.9% 17/215	*Grm2, Grm4*, *Htr4, Htr7, Rgs4*
	Serotonin receptor signaling	4.67E − 05	17.1% 7/41	*Slc6a4, Htr4, Htr7, Htr2a, Htr2c*
	Gi signaling	4.63E − 04	8.6% 10/116	*Grm2, Grm4, Gng3, Rgs4, Sos2*
**PND14**	Neuroprotective role of THOP1 in Alzheimer's disease	9.03E − 04	7.5% 3/40	*Nts, Pdyn, Prkag1*
	IGF‐1 signaling	1.14E − 03	4.2% 4/96	*Ctgf, Igfbp2, Igfbp4, Prkag1*
	EIF2 signaling	1.13E − 02	2.2% 4/183	*Rpl19, Rps2, Rps26, Rps29*
	mTOR signaling	1.22E − 02	2.1% 4/187	*Prkag1, Rps2, Rps26, Rps29*
	Notch signaling	1.40E − 02	5.3% 2/38	*Hes5, Mag*
**PND21**	Chondroitin sulfate degradation (Metazoa)	2.34E − 03	13.3% 2/15	*Cd44, Hyal1*
	Dermatan sulfate degradation (Metazoa)	2.67E − 03	12.5% 2/16	*Cd44, Hyal1*
	Growth hormone signaling	4.59E − 03	4.3% 3/69	*A2m, Fos, Igf2*
	Tight junction signaling	8.90E − 03	2.4% 4/167	*Cldn11, Fos, Myh11, Pvrl1*
	RhoGDI signaling	9.65E − 03	2.3% 4/171	*Cd44, Cdh7, Gng7, Rhog*
**PND35**	EIF2 signaling	2.94E − 05	3.3% 6/183	*Rpl19, Rpl41, Rps16, Rpl27a, Rps7*
	cAMP‐mediated signaling	6.86E − 04	2.3% 5/215	*Aplnr, Dusp1, Dusp4, Pde10a, Pde7b*
	G‐protein‐coupled receptor signaling	1.37E − 03	2.0% 5/251	*Aplnr, Dusp1, Dusp4, Pde10a, Pde7b*
	IL‐8 signaling	2.97E − 03	2.2% 4/182	*Fos, Ptgs2, Rhoj, Vegfb*
	ILK signaling	3.21E − 03	2.2% 4/186	*Fos, Ptgs2, Rhoj, Vegfb*
**PND70**	Growth hormone signaling	3.97E − 03	4.3% 3/69	*A2m, Fos, Igf2*
	EIF2 signaling	1.02E − 02	2.2% 4/183	*Rpl17, Rpl19, Rpl30, Rps2*
	Antigen presentation pathway	1.19E − 02	5.6% 2/36	*Psmb8, Tap2*
	Methionine Salvage II (Mammalian)	1.37E − 02	33.3% 1/3	*Bhmt*
	Role of tissue factor in cancer	1.43E − 02	2.7% 3/110	*Cyr61, Egr1, Hck*

Abbreviations: cAMP, cyclic adenosine monophosphate; EIF2, eukaryotic initiation factor 2; IGF‐1, insulin‐like growth factor 1; ILK, integrin‐linked kinase; IL‐8, interleukin 8; mTOR, mechanistic target of rapamycin; RhoGDI, Rho GDP‐dissociation inhibitor.

As we observed most expression changes between 5‐HTT^−/−^ and 5‐HTT^+/+^ rats at PND8 and GO and pathway analysis showed most reliable results in the subsequent analysis, we focused on gene expression data of PND8. For PND8, three of the top five most significantly enriched GO‐terms using DAVID were involved in neurotransmission (**transmission of nerve impulse**, **synaptic transmission,** and **cell–cell signaling**), with 24 genes that were linked to all three GO‐terms (Table S2). In addition, **neuropeptide signaling pathway** is in the top five GO‐terms, which contain peptides involved in a variety of neurodevelopmental processes like cell adhesion (e.g., *Gpr56*) and cell migration/axon guidance (e.g., *Sstr2*). Furthermore, **cell migration**, a process important in brain development, is in the top five GO‐terms and includes several factors and receptors involved in cell migration and axon guidance (e.g., *Ntn1, Nrp2, Dcc*, and *Sema3c*). Three of the top five pathways for PND8 using Ingenuity pathway analysis were involved in signal transduction; that is, **G‐protein‐coupled receptor signaling**, **cAMP‐mediated signaling,** and **Gi signaling**. The **G‐protein‐coupled receptor signaling** pathway included several serotonergic receptors (*Htr4, Htr7, Htr2a*, and *Htr2c*) and also showed substantial overlap with the **cAMP‐mediated signaling pathway** (15 genes) and the **Gi signaling pathway** (9 genes) (Table S3).

**FIGURE 3:**
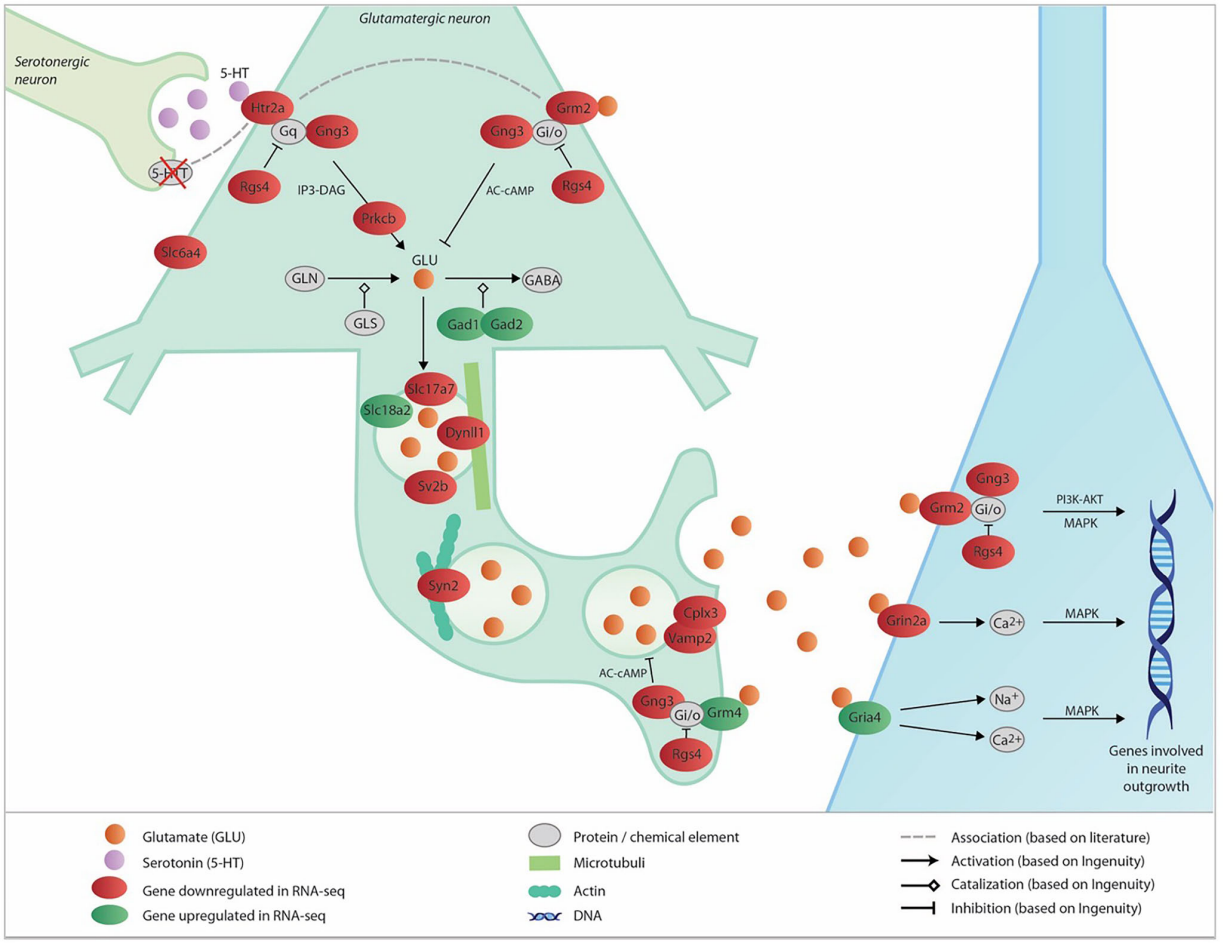
Potential neurotransmitter pathway affected by genetic 5‐HTT inactivation at PND8. Serotonin (5‐HT) is produced in the raphe nucleus and transported in vesicles to the axon terminals located in, among others, the prefrontal cortex (PFC). 5‐HT can be released from these vesicles into the synaptic space, where it can activate subtypes of receptors. 5‐HT_2A_, encoded by the *Htr2a* gene, is a Gq coupled 5‐HT receptor present on glutamatergic pyramidal cells in deep cortical layers (V and VI) (Vollenweider and Kometer 2010). Heterotrimeric G‐protein complexes are made up of alpha, beta and gamma (such as *Gng3*) subunits. RGS proteins, such as *Rgs4*, are able to deactivate G‐protein subunits (Neubig and Siderovski 2002). The most prominent pathway stimulated by the 5‐HT_2A_ receptor is the diacylglycerol (DAG) and inositol 1,4,5‐trisphosphate (IP3) pathway (IP3‐DAG) (Wischhof and Koch 2015) which activates Protein kinase C (encoded by *Prkcb*). Studies have shown that 5‐HT_2A_ receptor signaling is reduced in (adult) 5‐HTT^−/−^ mice (gray dotted line) (Qu et al. 2005; Rioux et al. 1999). Upon activation, 5‐HT_2A_ can trigger glutamate (GLU) release from the pyramidal cell (Vollenweider and Kometer 2010). GLU is synthesized by the conversion of glutamine (GLN) catalyzed by glutaminase (GLS) and packaged into synaptic vesicles by the vesicular glutamate transporters (VGLUTs; encoded by, among others, *Slc17a7*) (Rudy et al. 2015). Glutamic acid decarboxylases, encoded by *Gad1* and *Gad2*, catalyze the synthesis of gamma‐aminobutyric acid (GABA) from glutamate (Fehr et al. 2003). Long distance transport of synaptic vesicles, containing synaptic vesicular proteins (e.g., synaptic vesicle glycoprotein 2B encoded by *Sv2b* (Morgans et al. 2009)), occurs by kinesin and dynein (encoded by, among others, *Dynll1*) in the microtubule‐rich axon and vesicles then travel through the actin‐rich cortex at the nerve terminal on a myosin motor (Schlager and Hoogenraad 2009). Synapsins (encoded by, among others, *Syn2*) regulate the release of neurotransmitters by preventing vesicles from migrating to the presynaptic membrane by binding synaptic vesicles to actin (Bahler et al. 1990). The SNARE complex, composed of three membrane‐associated proteins (encoded by, among others, *Vamp2*), mediates the fusion of vesicles with the presynaptic membrane (Waites and Garner 2011). Complexin (encoded by, among others, *Cplx3*) acts as a positive regulator of synaptic vesicle exocytosis and binds selectively to the neuronal SNARE complex (Hu et al. 2002). Extracellular GLU can activate a variety of receptors. Each receptor can activate its own signal transduction pathway inside the postsynaptic neuron. mGlu2 receptors, encoded by the *Grm2* gene, are localized in the pre‐ and postsynaptic membrane (Gonzalez‐Maeso et al. 2008; Delille et al. 2013; Schoepp 2001). Activation of presynaptic mGlu2 receptors negatively modulates the release of glutamate by providing a feedback that prevents excessive glutamate release (Anwyl 1999; Scanziani et al. 1997) and regulates the release of other neurotransmitters (Cartmell and Schoepp 2000). Postsynaptic mGlu2 receptors can regulate neuronal excitability via the modulation of ion channels (Anwyl 1999). mGlu2 is a Gi/o coupled receptor that inhibits the adenylate cyclase (AC)—cyclic adenosine monophosphate (cAMP) pathway (Schoepp 2001) (presynaptic mGlu2) and can also activate the MAPK and PI3K‐AKT pathways (postsynaptic mGlu2) (Wischhof and Koch 2015; Niswender and Conn 2010). Studies have shown that mGlu2 can form a heterocomplex with the 5‐HT_2A_ receptor (Fribourg et al. 2011; Gonzalez‐Maeso et al. 2008) (gray dotted line). mGlu4, encoded by *Grm4*, is an autoreceptor localized at the active zone of boutons which inhibits the AC—cAMP pathway (Corti et al. 2002; Nicoletti et al. 2011). AMPA receptor (encoded by, among others, *Gria4*) activation can result in sodium and calcium influx. The intracellular signaling pathways used by AMPA receptors are not fully understood. One potential pathway is PI3K‐dependent activation of MAPK (Perkinton et al. 1999). NMDA receptor (encoded by, among others, *Grin2a*) activation results in an increase in calcium influx, which in turn activates MAPK signaling‐related cascades (Haddad 2005). Glutamate receptor signaling might affect transcription of genes involved in synapse formation and neurite outgrowth. For example, NMDA receptor signaling can influence netrin 1 (*Ntn1*) gene expression, which is a diffusible protein involved in cell migration and axon guidance and linked to 5‐HT (Bonnin et al. 2007; Toriumi et al. 2012). In addition, 5‐HTT (encoded by *Slc6a4*) and the vesicular monoamine transporter (encoded by *Slc18a2*) are transiently expressed in glutamatergic pyramidal neurons in early postnatal development, suggesting that 5‐HT can be taken up by glutamatergic neurons and can be released upon activation (Lebrand et al. 1998; Chen et al. 2015). Several genes involved in vesicular transport and downstream signaling pathways shown in this network are downregulated in the 5‐HTT^−/−^ rats, suggesting reduced synaptic transmission. 5‐HT, 5‐hydroxytryptamine.

On the basis of the results of DAVID (genes linked to neurotransmission), ingenuity (genes linked to signal transduction), and systematic literature search, we built a potential network involving 5‐HT and glutamate signaling in the mPFC (Figure 3). Among the receptors showing differential expression at PND8 is the 5‐HT receptor gene *Htr2a*, which upon activation can stimulate glutamate release (Vollenweider and Kometer 2010). Several genes encoding for glutamatergic receptors (*Grm2, Grm4, Gria4, Grin2a)* were also differentially expressed in the mPFC. In addition, certain genes involved in synthesis (*Ddc*), vesicular transport (*Slc17a7* (Rudy et al. 2015)*, Sv2b* (Morgans et al. 2009)*, Dynll1* (Schlager and Hoogenraad 2009)*, Syn2* (Bahler et al. 1990)), and release (*Vamp2* (Waites and Garner 2011)*, Cplx3* (Hu et al. 2002)) of neurotransmitters and genes involved in downstream signal transduction pathways (*Rgs4* (Neubig and Siderovski 2002)*, Gng3* (Clapham and Neer 1997)*, Prkcb* (Wischhof and Koch 2015)) were downregulated in the 5‐HTT^−/−^ mPFC, suggesting an overall decrease in neurotransmission. Glutamic acid decarboxylases, encoded by *Gad1* and *Gad2* (Fehr et al. 2003), catalyzing the synthesis of gamma‐aminobutyric acid (GABA) from glutamate, were upregulated in the 5‐HTT^−/−^ mPFC.

### 3.4 RNA‐Seq Validation by RT‐qPCR

**FIGURE 4:**
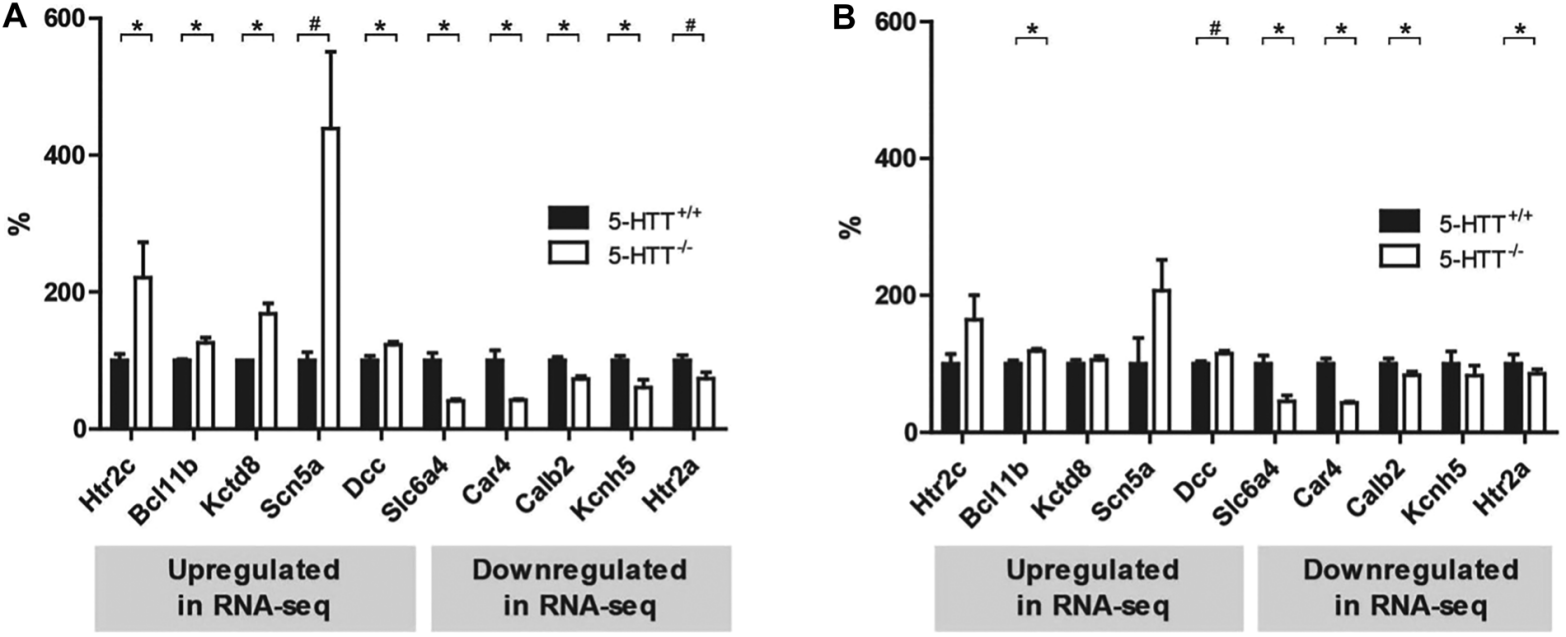
Medial prefrontal cortex RNA expression levels in 5‐HTT^−/−^ and 5‐HTT^+/+^ rats by RT‐qPCR. Validation of RNA‐seq results by quantitative RT‐PCR (RT‐qPCR) analysis in RNA‐seq samples (A) and in independent biological replicates (B). RT‐qPCR analysis was performed on medial prefrontal cortex RNA of 5‐HTT^−/−^ and 5‐HTT^+/+^ rats sacrificed at PND8. On the basis of RNA‐seq data, five genes that were upregulated (left side in figure) and five genes that were downregulated (right side in figure) in 5‐HTT^−/−^ samples were compared to the 5‐HTT^+/+^ samples that were selected for validation. Data are normalized for *Gabbr1* RNA levels and are presented as mean + SEM of relative gene expression (% of 5‐HTT^+/+^ group). *p < 0.05; ^#^ *p* < 0.1.

To validate the RNA‐seq experiment at PND8, we selected five up‐ and five downregulated genes for RT‐qPCR validation in the RNA‐seq samples (five per genotype) and in five independent samples per genotype. Using the RNA‐seq samples, four out of the five genes downregulated in 5‐HTT^−/−^ rats by RNA‐seq were significantly downregulated when assessed by RT‐qPCR as well (*Slc6a4* (*t*_(1,4.39)_ = 5.20; *p* < 0.05); *Car4* (*t*_(1,4.07)_ = 3.90; *p* < 0.05); *Calb2* (*t*_(1,7)_ = 3.93; *p* < 0.05); *Kcnh5* (*t*_(1,7)_ = 3.17; *p* < 0.05)); and *Htr2a* showed a trend (*t*_(1,7)_ = 2.18; *p* < 0.1). Four out of the five genes upregulated in the 5‐HTT^−/−^ rats by RNA‐seq were significantly upregulated when validated by RT‐qPCR (*Htr2c* (*t*_(1,7)_ = 2.60; *p* < 0.05); *Bcl11b* (*t*_(1,3.29)_ = 3.21; *p* < 0.05); *Kctd8* (*t*_(1,5)_ = 3.80; *p* < 0.05); *Dcc* (*t*_(1,7)_ = 2.83; *p* < 0.05); and *Scn5a* showed a trend (*t*_(1,3.07)_ = 3.02; *p* < 0.06) in the right direction (Figure 4A). We repeated the RT‐qPCR in independent samples. *Slc6a4* (*t*_(1,7)_ = 5.37; *p* < 0.05), *Car4* (*t*_(1,3.22)_ = 5.95; *p* < 0.05), and *Htr2a* (*t*_(1,7)_ = 2.62; *p* < 0.05) were all significantly downregulated in the 5‐HTT^−/−^ rats. *Bcl11b* (*t*_(1,7)_ = 5.51; *p* < 0.05) was significantly upregulated, and *Dcc* showed a trend for upregulation in the 5‐HTT^−/−^ rats (*t*_(1,7)_ = 2.04; *p* < 0.1). The other genes do not reach the threshold of significance, but all genes showed a change in the same direction as observed in the RNA‐seq experiment (Figure 4B).

### 3.5 Genome‐Wide DNA (Hydroxy)Methylation

5mC and 5hmC levels were measured in mPFC DNA of five samples per genotype for each time point. No significant differences were found in 5mC levels between 5‐HTT^−/−^ and 5‐HTT^+/+^ rats (Figure S4A). For the 5hmC levels, we found a significant increase in 5‐HTT^−/−^ compared to 5‐HTT^+/+^ rats at PND35 (*t*_(1,8)_ = 6.29; *p* < 0.05) (Figure S4B). In addition, 5hmC levels increased during development from 0.38% 5hmC/G at PND8 to 0.71% 5hmC/G at PND70 in both genotypes. Validation of the increase in 5hmC levels at PND35 was performed in an independent group of biological replicates (Group 3, see Section 2). In this group, 5hmC levels in mPFC tissue were again increased in the 5‐HTT^−/−^ rats compared to the 5‐HTT^+/+^ rats (*t*_(1,18)_ = 2.28; *p* < 0.05) (Figure S4B). No significant expression changes were observed in genes involved in DNA (hydroxy)methylation (*Tet* and *Dnmt* genes) at PND35, nor at the other time points (Figure S3).

## 4 Discussion

In this study, we demonstrate that a lack of functional 5‐HTT affects postnatal gene expression in the mPFC particularly during the first postnatal week (PND8, earliest time point measured), with 615 differentially expressed genes between 5‐HTT^−/−^ and 5‐HTT^+/+^ rats for PND8 and less than 140 differentially expressed genes for the other time points (PND14, 21, 35, and 70). GO analysis showed that genes differentially expressed in the mPFC of 5‐HTT^−/−^ rats at PND8 are mainly involved in neurotransmission and neurodevelopmental processes, whereas GO‐terms enriched at other time points are less specific for neuronal‐related processes.

Expression of *Slc6a4* (encoding for 5‐HTT itself) is in general low in the mPFC, because the cell soma (where most of the RNA translation takes place (Kim and Jung 2015)) of the serotonergic neurons is located in the raphe nuclei, whereas the axons (containing 5‐HTT protein) reach the mPFC. We do see higher levels of *Slc6a4* mRNA at early postnatal time points (PND8 and PND14), which can be explained by the fact that at early postnatal stages 5‐HTT is not only expressed by serotonergic neurons from the raphe nuclei, but also by glutamatergic neurons in, among others, the mPFC (see Section 1) (Homberg et al. 2010). We see a decrease in the expression of the mutated *Slc6a4* gene in the 5‐HTT^−/−^ mPFC at PND8, most likely because the RNA containing the point mutation is degraded by nonsense‐mediated decay (Keeling et al. 2004).

PCA and cluster analysis demonstrated that 5‐HTT^+/+^ and 5‐HTT^−/−^ samples cluster together per age, showing that the differences between the developmental time points were larger than the differences between genotypes. The stronger difference between time points is not surprising given the tremendous changes in the brain that occur from the early postnatal period up to adulthood compared to the relatively mild phenotype observed in the 5‐HTT^−/−^ rats. We also observed a clear gene expression separation between early (PND8, 14, and 21) and late (PND35 and 70) ages in the PCA, possibly indicating a genetic switch from PFC development at early postnatal life to maintenance of neural networks in adulthood. This shift from development to maintenance between PND21 and 35 was shown in our 5‐HTT^+/+^ PFC samples (Kroeze et al. 2017) and coincides with the sharp increase in synapse formation during early brain development, which stabilizes around adolescence (Kolb et al. 2012; Togashi et al. 2009). However, per time point, we do see differences in gene expression between 5‐HTT^+/+^ and 5‐HTT^−/−^ mPFC samples. There are 123 genes showing differential expression at more than one time point, among others the myelin‐related genes *Cldn11*, *Plp1*, *Mbp*, and *Mag*. Overall, there is a decrease in expression of myelin genes in 5‐HTT^−/−^ compared to 5‐HTT^+/+^ rats, potentially resulting in decreased or aberrant myelin sheaths, which fits with our previous work showing that a lack of functional 5‐HTT (blocking 5‐HTT by selective serotonin reuptake inhibitors) early in life results in downregulation of genes linked to myelin (Kroeze et al. 2015) as well as with work of others showing a disturbed myelin sheath formation after blocking 5‐HTT perinatally (Simpson et al. 2011). Why we observe an opposite effect at PND14 is unclear. In both 5‐HTT^−/−^ and 5‐HTT^+/+^ rats, the highest expression of myelin‐related genes was observed around PND21. It has been documented that myelin formation in the brain starts around PND10 in the rat and that the maximum rate of myelin accumulation occurs around PND20. Myelin accumulation does continue into adulthood, albeit at a decreasing rate (Downes and Mullins 2014; Doretto et al. 2011). This matches with the peak of mRNA expression we see around PND20.

Most differences in mPFC gene expression between 5‐HTT^−/−^ and 5‐HTT^+/+^ rats were found at PND8. At PND8, we found mainly GO‐terms and pathways involved in cell migration, neurotransmission, and signal transduction (see Tables 1 and 2). **Cell migration**, a process important for proper formation of cell layers and cell connections in the brain, is in line with literature showing that 5‐HT is involved in the migration of neurons and connectivity in the brain (Gaspar et al. 2003; Riccio et al. 2009; Migliarini et al. 2013). In addition, studies have shown that cell migration and connectivity are disturbed in 5‐HTT^−/−^ mice and S‐allele carriers, respectively (Riccio et al. 2009; Wiggins et al. 2012). Furthermore, cell migration is affected in 5‐HT‐related neuropsychiatric disorders such as autism spectrum disorder and bipolar disorder (Ishii et al. 2016). In the neurotransmission‐related GO‐terms and signal transduction pathways enriched at PND8, several genes involved in synthesis and transmission of glutamate were found. The differentially expressed 5‐HT_2A_ receptor is a serotonergic receptor present on glutamatergic pyramidal cells in deep cortical layers (V and VI) in the PFC, which, upon activation, can trigger glutamate release from the pyramidal cells (Figure 4) (Vollenweider and Kometer 2010). In addition, studies have shown that 5‐HTT (encoded by *Slc6a4*) and the vesicular monoamine transporter (encoded by *Slc18a2*), which are both differential expressed at PND8, are transiently expressed on glutamatergic pyramidal neurons in early postnatal development, suggesting that 5‐HT can be taken up by glutamatergic neurons and can be released upon activation (Lebrand et al. 1998; Chen et al. 2015). Extracellular glutamate can activate glutamate receptors on layer V pyramidal cells (Vollenweider and Kometer 2010). The downregulation of several genes involved in vesicular transport of neurotransmitters and in downstream signaling pathways in the 5‐HTT^−/−^ rats suggests a reduction in synaptic transmission in these rats. During postnatal development, NMDA receptors (encoded by, among others, *Grin2a*) and AMPA receptors (encoded by, among others, *Gria4*) are involved in neurite outgrowth and the formation, stabilization, maturation, and elimination of synapses (Zhang and Poo 2001; Ewald and Cline 2009; Sin et al. 2002; Bolton et al. 2000). Although the involvement of mGlu receptors (encoded by, among others, *Grm2* and *Grm4*) in such processes is largely unknown, it has been suggested that they may also participate in synapse‐stabilizing responses (Miskevich et al. 2002). This implies that altered expression of these glutamate receptors in the early postnatal PFC, as shown in our RNA‐seq experiment, results in dysregulation of neurite outgrowth and synaptogenesis. Whether this dysregulation at PND8 can also result in neuropsychiatric disorders later in life is not clear. However, it has been shown that alterations in the serotonergic 5‐HT_2A_ and glutamatergic receptors are associated with neuropsychiatric disorders. Interactions between *Htr2a* polymorphisms and *Slc6a4* polymorphisms or SSRI treatment (blocking *Slc6a4*) were shown to predict treatment response in anxiety and depression (Kato et al. 2009; Lohoff et al. 2013; Choi et al. 2005; McMahon et al. 2006; Kato et al. 2006). Furthermore, studies showed that 5‐HT_2A_ receptor signaling is reduced in adult 5‐HTT^−/−^ mice which show depression‐like behavior (Qu et al. 2005; Rioux et al. 1999). Altered *Grin2a* expression is linked to depression‐like behavior in rats (Sun et al. 2013). In addition, the mGlu2 receptor, encoded by *Grm2*, has been shown to be altered in both animal models of depression and in postmortem brain tissue of subjects with major depressive disorder (Feyissa et al. 2010; Matrisciano et al. 2008). Recent studies have shown that 5‐HT_2A_ and mGlu2 receptors can form a functional receptor heterocomplex, which is shown to be dysregulated in the frontal cortex of schizophrenic patients (Wischhof and Koch 2015; Fribourg et al. 2011; Gonzalez‐Maeso et al. 2008; Kurita et al. 2012). Of the genes differentially regulated at PND8, Grm2, Htr2a, and Grin2a were also found to be differentially regulated in our previous 5‐HTT^+/+^ PFC samples (Kroeze et al. 2017), suggesting that these genes transcriptionally regulated by age are affected by 5‐HTT expression.

The differences found in gene expression between 5‐HTT^−/−^ and 5‐HTT^+/+^ rats might influence (neuro)development and behavior. We previously demonstrated that the 5‐HTT^−/−^ rats show a delay in development (Kroeze et al. 2016). One of the main differences we observed was reduced motor coordination and muscle strength in the 5‐HTT^−/−^ rats in infancy. The underlying mechanism causing this aberrant development is still unknown, but it might be caused by disturbed gene expression. In our Ingenuity Top Diseases and Bio Functions list from PND8 (Table S4), we see the highest enrichment for genes linked to **neurological diseases** and also a high enrichment for **skeletal and muscular disorders**. Within the **neurological diseases** category, we see only motor‐ and muscle‐related diseases in the top three of the list, namely, **seizure disorder**, **movement disorders,** and **dyskinesia** (Table S5). This suggests that several differentially expressed genes at PND8 might be associated with the defects in muscle and motor function we found in young 5‐HTT^−/−^ rats. From the genes we used for qPCR validation, *Htr2a, Htr2c, Scn5a, Bcl11b, Dcc, Car4*, and *Slc6a4* are linked to the Ingenuity term **movement disorders** (Al Hadithy et al. 2008; Desplats et al. 2008; Meneret et al. 2015; Segman et al. 2001; Awad et al. 2001; Hedera et al. 2013; Moya et al. 2013). Studies have shown that regulation of the expression of the serotonergic 5‐HT_2A_ receptor gene (*Htr2a*) can influence motor control (Paquette et al. 2013; Paquette and Marsit 2014). *Htr2a* promoter methylation, associated with reduced expression, is linked to a reduced quality of movement in infants (Paquette et al. 2013). In addition, polymorphisms in the *Htr2a* gene are linked to movement‐related diseases like tardive dyskinesia (Segman et al. 2001) and Parkinson's disease (Lee et al. 2012). We have to keep in mind that we focused on expression changes in the mPFC, which is not a main motor control region (Siddiqui et al. 2008). Nonetheless, gene expression changes observed in the mPFC do not have to be PFC‐specific. More functional research (e.g., in muscles or the motor cortex) is needed to elucidate the link between the expression of these genes and the behavioral differences found in 5‐HTT^−/−^ rats.

We have to keep in mind that 5‐HTT expression starts prenatally. Therefore, the expression differences found in the 5‐HTT^−/−^ rats postnatally can be a secondary effect of the changes occurring prenatally. Gene expression at postnatal stage can be different due to a different cell composition of the punch area caused by prenatal effects on developmental processes, for example, cell migration. In addition. The expression differences in the 5‐HTT^−/−^ rats at postnatal time points can be a compensatory mechanism for the expression changes occurring prenatally. Genome‐wide gene expression data from prenatal stages would give valuable new insights. Finally, we used whole mPFC tissue containing several different cell types, not only neurons but also microglia, astrocytes, and oligodendrocytes. Given that the differentially regulated genes we observed at P8 are different from the differentially regulated genes found at P7 specifically in the 5‐HTT‐expressing neurons in mPFC of 5‐HTT^−/−^ mice (Soiza‐Reilly et al. 2019), our findings likely relate to effects of 5‐HTT knockout on interactions among different types of neurons and support cells rather than exclusively 5‐HTT‐expressing glutamatergic neurons.

We did not find any differences in global 5mC in the mPFC, but we found an increase in 5hmC in the mPFC across development. This is consistent with previous findings showing that 5hmC is highly abundant in the brain (Nestor et al. 2012) and levels increase during pre‐ and postnatal neurodevelopment (Szulwach et al. 2011; Hahn et al. 2013). DNA methylation plays an important role in neurogenesis, neuronal maturation, and plasticity (Watanabe et al. 2006; Feng et al. 2010) and is linked to 5‐HT‐related diseases such as depression and anxiety (Murphy et al. 2015; Zhang et al. 2015). It has previously been demonstrated that 5‐HT induces methylation of a conserved CpG island in the promoter region of the *CREB2* gene, serving synaptic plasticity (Rajasethupathy et al. 2012). Furthermore, we found reduced global 5‐hmC levels (Shan et al. 2014) and altered methylation of the corticotrophin‐releasing factor gene (van der Doelen et al. 2015) in the amygdala of 5‐HTT^−/−^ rats at adulthood. We observed a significant increase in global 5hmC in 5‐HTT^−/−^ compared to 5‐HTT^+/+^ rats at PND35. However, the difference in 5hmC between 5‐HTT^−/−^ and 5‐HTT^+/+^ rats at PND35 does not coincide with differences in gene expression; we did not observe a sudden increase in the number of differentially expressed genes between the 5‐HTT^−/−^ and 5‐HT^+/+^ rats at PND35. Moreover, no differences in *Tet1, Tet2*, and *Tet3* expression were observed (Figure S3). As the critical period for development of the inhibitory system in the prefrontal cortex occurs during adolescence (Larsen and Luna 2018), it has been suggested that 5‐HTT gene ablation affects the critical periods for sensory cortical regions earlier in life (Homberg et al. 2024), and critical periods are associated with increased sensitivity for environmental stimuli, and it is possible that the increase in 5hmC specifically at P35 reflects the impact of environmental stimuli on prefrontal cortex maturation. Further research is needed to unravel the exact reason and consequences of increased 5hmC during adolescence. In addition, it should be noted that we measured 5(h)mC at a global level, which means that methylation changes on specific genes can still be present despite the lack of an overall effect.

This study comes with some limitations. First, we studied only male rats. Therefore, findings may not generalize to female rats. Second, follow‐up research is required to validate the differently expressed genes at protein level and to identify the cellular location of the proteins. Finally, causal studies are needed to link gene expression changes to behavioral phenotypes of the 5‐HTT^−/−^ rats.

In conclusion, we provide a comprehensive overview of differentially expressed genes in the mPFC of 5‐HTT^−/−^ rats compared to 5‐HTT^+/+^ rats across postnatal development. We showed that reduced functional 5‐HTT expression affects gene expression in the mPFC mainly during early postnatal life (PND8). These changes in gene expression can affect neurodevelopmental processes resulting in aberrant brain wiring which can influence behavior during infancy but also can result in lifelong consequences on brain functioning, potentially influencing vulnerability to neuropsychiatric disorders in adolescence and adulthood. Although mainly adult rats have been used to unravel the mechanisms associated with 5‐HT‐related neuropsychiatric disorders, we propose that studying early life stages might give more insight in the developmental dysregulation that might predispose to 5‐HT‐related neuropsychiatric disorders in adulthood.

## Author Contributions

Yvet Kroeze, Judith R. Homberg, and Huiqing Zhou contributed to the study conception and design. Judith R. Homberg, Sharon M. Kolk, Hans van Bokhoven, and Huiqing Zhou supervised the project. Material preparation, data collection, and analysis were performed by Yvet Kroeze, Martin Oti, Roel H. M. Cooijmans, and Leonie I. Kroeze. Anthonieke Middelman coordinated the animal breeding. Ellen van Beusekom supported the sample preparations. The first draft of the article was written by Yvet Kroeze and all authors commented on previous versions of the article. All authors read and approved the final article.

## Ethics Statement

The animal experiments were approved by the Animal Ethics Committee of the Radboud University. All experiments were carried out according to the guidelines for the Care and Use of Mammals in Neuroscience and Behavioral Research (National Research Council 2003), the principles of laboratory animal care, as well as the Dutch law concerning animal welfare.

## Conflicts of Interest

The authors declare no conflicts of interest.

## Supporting information




**Table S1** Primers used for quantitative RT‐PCR.
**Table S2** Differentially expressed genes (PND8) linked to three GO‐terms.
**Table S3** Differentially expressed genes (PND8) linked to the G‐protein‐coupled receptor signaling pathway in Ingenuity.
**Table S4** Top Disease and Bio Functions affected by 5‐HTT^−/−^ in the medial prefrontal cortex.
**Table S5** Top Neurological Diseases affected by 5‐HTT^−/−^ in the medial prefrontal cortex.
**Table S6** Heatmap gene expression data.
**Figure S1** The distribution of coefficients of variation across all genes.
**Figure S2** Comparison of 5‐HTT^+/+^ and 5‐HTT^−/−^ samples to the reference genome and principal component analysis.
**Figure S3** Expression of genes involved in DNA methylation.
**Figure S4** 5(h)mC measurements in medial prefrontal cortex DNA of 5‐HTT^−/−^ and 5‐HTT^+/+^ rats.

**Table S2** Diff expressed genes and GO analysis.

**Table S6** Heatmap_Matrix_WT_KO_ZScores_CoV0_3_.

## Data Availability

The datasets generated during and/or analyzed during the current study are available in the GEO repository under number GSE79909.
