# Supplementary material for: Genetic Inactivation of the Serotonin Transporter Dysregulates Expression of Neurotransmission Genes and Genome‐Wide DNA Methylation Levels in the Medial Prefrontal Cortex of Male Rats During Postnatal Development
Source: Dev Neurobiol. 2025 Jun 8;85(3):e22973. doi: 10.1002/dneu.22973 (PMC12146603; doi:10.1002/dneu.22973)
Supplement: Supplementary file 1 — Table S1 Primers used for quantitative RT‐PCR. Table S2 Differentially expressed genes (PND8) linked to three GO‐terms. Table S3 Differentially expressed genes (PND8) linked to the G‐protein‐coupled receptor signaling pathway in Ingenuity. Table S4 Top Disease and Bio Functions affected by 5‐HTT−/− in the medial prefrontal cortex. Table S5 Top Neurological Diseases affected by 5‐HTT−/− in the medial prefrontal cortex. Table S6 Heatmap gene expression data. Figure S1 The distribution of coefficients of variation across all genes. Figure S2 Comparison of 5‐HTT+/+ and 5‐HTT−/− samples to the reference genome and principal component analysis. Figure S3 Expression of genes involved in DNA methylation. Figure S4 5(h)mC measurements in medial prefrontal cortex DNA of 5‐HTT−/− and 5‐HTT+/+ rats. [file DNEU-85-0-s001.docx]

**Supplementary information**

1. Table S1: Primers used for quantitative RT-PCR
2. Table S2: Differentially expressed genes (PND8) linked to three GO-terms
3. Table S3: Differentially expressed genes (PND8) linked to the G protein coupled receptor signaling pathway in Ingenuity
4. Table S4: Top Disease and Bio Functions affected by 5-HTT^-/-^ in the medial prefrontal cortex
5. Table S5: Top Neurological Diseases affected by 5-HTT^-/-^ in the medial prefrontal cortex
6. Table S6: Heatmap gene expression data
7. Figure S1: The distribution of coefficients of variation across all genes
8. Figure S2: Comparison of 5-HTT^+/+^ and 5-HTT^-/-^ samples to the reference genome and principal component analysis.
9. Figure S3: Expression of genes involved in DNA methylation
10. Figure S4: 5(h)mC measurements in medial prefrontal cortex DNA of 5-HTT^-/-^ and 5-HTT^+/+^ rats.

**Table S1**: Primers used for quantitative RT-PCR

| **Gene symbol** | **Ensembl number** | **Forward primer** | **Reverse primer** |
| --- | --- | --- | --- |
| *Htr2c* | ENSRNOG00000030877 | ACGCACGTGGTGTTATTCAC | ATGAAGATTGCCATCGTTTG |
| *Slc32a1* | ENSRNOG00000015393 | CACGACAAACCCAAGATCAC | GAGGATGGCGTAGGGTAGAC |
| *Kctd8* | ENSRNOG00000026472 | GACGACAAGATCTGGAGCAG | GCAGGAAGTCCCACTCTCAC |
| *Scn5a* | ENSRNOG00000015049 | TTCTGGATGATGTCGGAGAG | TGCGCCACTATTACTTCACC |
| *Dcc* | ENSRNOG00000033099 | GTCTGGGAGAGGAACCTCAG | CAGACACCATAAACCGGATG |
| *Slc6a4* | ENSRNOG00000003476 | CTGTGGGTGTTTCAGGAGTG | GCTACTGCATAGGGATGTCG |
| *Car4* | ENSRNOG00000002916 | TCGTTGGCTATGACCAAAAG | CAGGTGTAACTGTATGGCCTTG |
| *Calb2* | ENSRNOG00000016977 | GCCATCTCAATTTTCCCATC | AAGGCCAGGAAAGGTTCTG |
| *Kcnh5* | ENSRNOG00000009542 | CACATACCAGGAGCACAAGG | CAATGCCTTTGAAAATGTGG |
| *Htr2a* | ENSRNOG00000010063 | GTTTCCTTGTCATGCCTGTG | TGATGGATGCCGTAGAAAAG |
| *Gabbr1* | ENSRNOG00000000774 | TTTTCAGCCGCTTGGTTAG | ACATCACCACGGAGATTGTC |

**Table S2**: Differentially expressed genes (PND8) linked to three GO-terms:

Transmission of nerve impulse, cell - cell signaling and synaptic transmission

| **Gene** | **Log2 Ratio** | **Fold change** | **p-value** | **FPKM** |
| --- | --- | --- | --- | --- |
| *Cartpt* | 0.90 | 1.86 | 5.00E-05 | 42.233 |
| *Cplx3* | -0.73 | 0.60 | 1.00E-04 | 2.515 |
| *Slc6a4* | -1.88 | 0.27 | 5.00E-05 | 1.479 |
| *Sv2b* | -0.31 | 0.81 | 9.00E-04 | 117.867 |
| *Pnoc* | 0.76 | 1.69 | 8.00E-04 | 5.065 |
| *Vamp2* | -0.27 | 0.83 | 4.65E-03 | 80.164 |
| *Sptbn2* | -0.26 | 0.83 | 5.35E-03 | 195.726 |
| *Shc3* | -0.42 | 0.75 | 1.00E-04 | 38.991 |
| *Grm2* | -0.75 | 0.59 | 5.00E-05 | 2.862 |
| *Syn2* | -0.32 | 0.80 | 1.15E-03 | 47.932 |
| *P2rx2* | -0.48 | 0.72 | 8.80E-03 | 4.430 |
| *Pdyn* | 0.63 | 1.54 | 5.00E-05 | 6.293 |
| *Thbs2* | 0.91 | 1.88 | 5.00E-05 | 1.781 |
| *Hap1* | 0.42 | 1.34 | 5.00E-05 | 66.095 |
| *Gria4* | 0.28 | 1.21 | 4.80E-03 | 55.424 |
| *Gad2* | 1.14 | 2.20 | 5.00E-05 | 53.588 |
| *Dynll1* | -0.28 | 0.82 | 3.20E-03 | 337.976 |
| *Grin2a* | -0.34 | 0.79 | 3.10E-03 | 10.231 |
| *Gad1* | 0.70 | 1.63 | 5.00E-05 | 61.382 |
| *Gabrg1* | 0.27 | 1.21 | 9.60E-03 | 45.799 |
| *Slc17a7* | -0.38 | 0.77 | 2.00E-04 | 165.800 |
| *Chrm1* | -0.56 | 0.68 | 5.00E-05 | 17.832 |
| *Slc22a3* | -0.76 | 0.59 | 5.00E-05 | 8.515 |
| *Slc5a7* | 0.45 | 1.36 | 1.15E-03 | 4.518 |

**Table S3**: Differentially expressed genes (PND8) linked to the G protein coupled receptor signaling pathway in Ingenuity

| **Gene** | **Log2 Ratio** | **Fold change** | **p-value** | **FPKM** | **Ingeniuty pathway** |
| --- | --- | --- | --- | --- | --- |
| *Atf4* | -0.31 | 0.80 | 9.50E-04 | 187.949 | GPCR, cAMP |
| *Chrm1* | -0.56 | 0.68 | 5.00E-05 | 17.832 | GPCR, cAMP |
| *Dusp4* | 0.53 | 1.44 | 4.00E-04 | 13.146 | GPCR, cAMP |
| *Grm2* | -0.75 | 0.59 | 5.00E-05 | 2.862 | GPCR, cAMP, GI |
| *Grm4* | 0.60 | 1.51 | 3.70E-03 | 3.098 | GPCR, cAMP, GI |
| *Hrh3* | -0.47 | 0.72 | 4.30E-03 | 4.836 | GPCR, cAMP, GI |
| *Htr4* | 0.78 | 1.71 | 1.25E-03 | 3.186 | GPCR, cAMP, SERT |
| *Htr7* | 0.69 | 1.61 | 5.00E-05 | 5.535 | GPCR, cAMP, SERT |
| *Htr1f* | 1.03 | 2.04 | 4.00E-03 | 1.374 | GPCR, cAMP, GI |
| *Mc4r* | 0.97 | 1.96 | 6.50E-04 | 2.759 | GPCR, cAMP |
| *Npr3* | -0.30 | 0.81 | 9.25E-03 | 25.496 | GPCR, cAMP, GI |
| *Npy1r* | -0.38 | 0.77 | 8.00E-04 | 18.629 | GPCR, cAMP, GI |
| *Oprk1* | 1.00 | 1.99 | 5.00E-05 | 2.150 | GPCR, cAMP, GI |
| *Pde8b* | 0.56 | 1.48 | 4.50E-04 | 5.496 | GPCR, cAMP |
| *Rgs4* | -0.52 | 0.70 | 5.00E-05 | 146.125 | GPCR, cAMP, GI |
| *Htr2a* | -0.38 | 0.77 | 1.10E-03 | 11.042 | GPCR, SERT |
| *Htr2c* | 1.47 | 2.76 | 5.00E-05 | 19.846 | GPCR, SERT |
| *Sos2* | -0.40 | 0.76 | 1.00E-04 | 16.180 | GPCR, GI |
| *Adra1d* | -0.70 | 0.62 | 5.00E-05 | 2.751 | GPCR |
| *Prkcb* | -0.28 | 0.82 | 1.80E-03 | 186.166 | GPCR |

Abbreviations: GPCR = G protein coupled receptor signaling, cAMP = cAMP mediated signaling and SERT = serotonin mediated signaling

**Table S4**: Top Disease and Bio Functions affected by 5-HTT^-/-^ in the medial prefrontal cortex

|  | **Pathway** | **Top PValue** | **Genes** |
| --- | --- | --- | --- |
| **PND8** | Neurological Disease | 8.13E-14 | 142 |
|  | Psychological Disorders | 2.03E-12 | 103 |
|  | Hereditary Disorder | 2.37E-12 | 71 |
|  | Skeletal and Muscular Disorders | 2.37E-12 | 106 |
|  | Cancer | 1.83E-11 | 409 |

|  | **Pathway** | **PValue** | **Genes (nr and examples)** |
| --- | --- | --- | --- |
| **PND8** | Seizure disorder | 8.13E-14 | *46 (e.g. Kcnh3, Kcnj9, Galr1, Gng3, Pnoc)* |
|  | Movement Disorders | 1.55E-13 | *69 (e.g. Htr2a, Htr2c, Bcl11b, Dcc, Scn5a)* |
|  | Dyskinesia | 1.34E-12 | *44 (e.g. Hpca, S100a10, Grin2a, Sst, Tnnt1)* |
|  | Seizures | 1.38E-12 | *40 (e.g. Kcnh3, Kcnj9, Galr1, Gng3, Pnoc)* |
|  | Disorder of basal ganglia | 2.03E-12 | *50 (e.g. Syn2a, Penk, Gpr88, Ptpn3, Hpca)* |

**Table S5**: Top Neurological Diseases affected by 5-HTT^-/-^ in the medial prefrontal cortex

**Table S6**: Data underlying the heatmap presented in Figure 2

**Figure S1**

**Figure S1.** The distribution of coefficients of variation across all genes

**Figure S2**

**
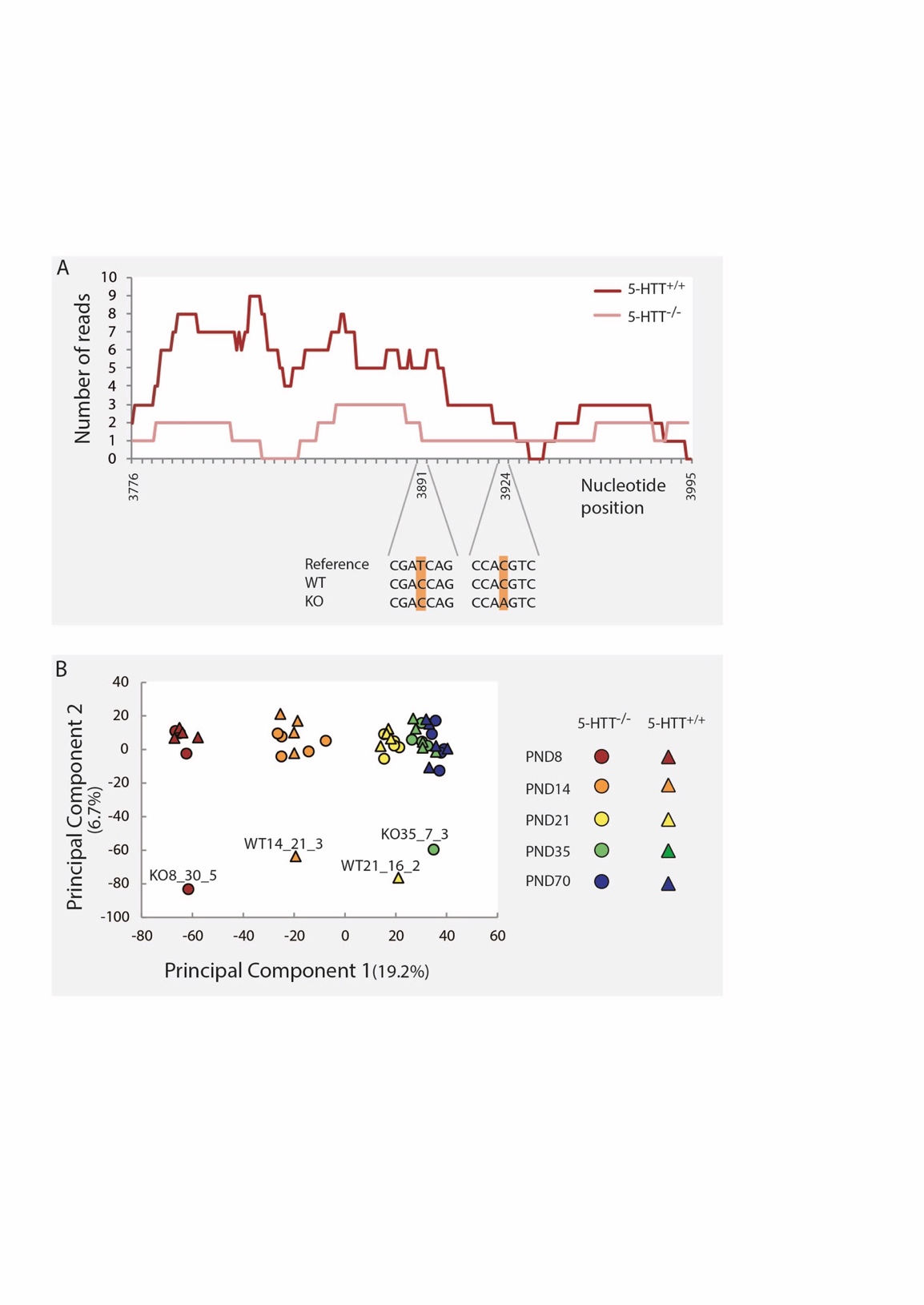
**

**Figure S2. Comparison of 5-HTT^+/+^ and 5-HTT^-/-^ samples to the reference genome and principal component analysis**. A: Comparison of reads from 5-HTT^+/+^ and 5-HTT^-/-^ samples to the reference *Slc6a4* gene. The number of reads that span a position in the third exon of the *Slc6a4* gene is depicted on the y-axis in the graph. The x-axis depicts the nucleotide position based on the ensembl gene ENSRNOG0000003476. 5-HTT^+/+^ is indicated by the red line and 5-HTT^-/-^ is indicated by the pink line. Sequences deviating from ENSRNOG0000003476 (based on the Brown Norway rat, rn4 assembly [72]) are shown under the graph. The highlighted nucleotides correspond to the locations in the graph. B: Principal component analysis (PCA). PCA was performed on log10 transformed Fragments Per Kilobase per Million mapped reads (FPKM) values (a pseudocount of 0.01 was added to avoid infinite values) and small genes were excluded. The axes are labelled by the principal component they represent and the percentage of explained variance by the principal component. Circles represent 5-HTT^-/-^ samples and triangles represent 5-HTT^+/+^ samples. Red represents PND8, orange represents PND14, yellow represents PND21, green represents PND35 and blue represents PND70. Explanation sample numbers: WT (wild-type) = 5-HTT^+/+^, KO (knockout) = 5-HTT^-/-^. Genotype is followed by postnatal day, litter number and number of the rat in the litter.

**Figure S3**

**
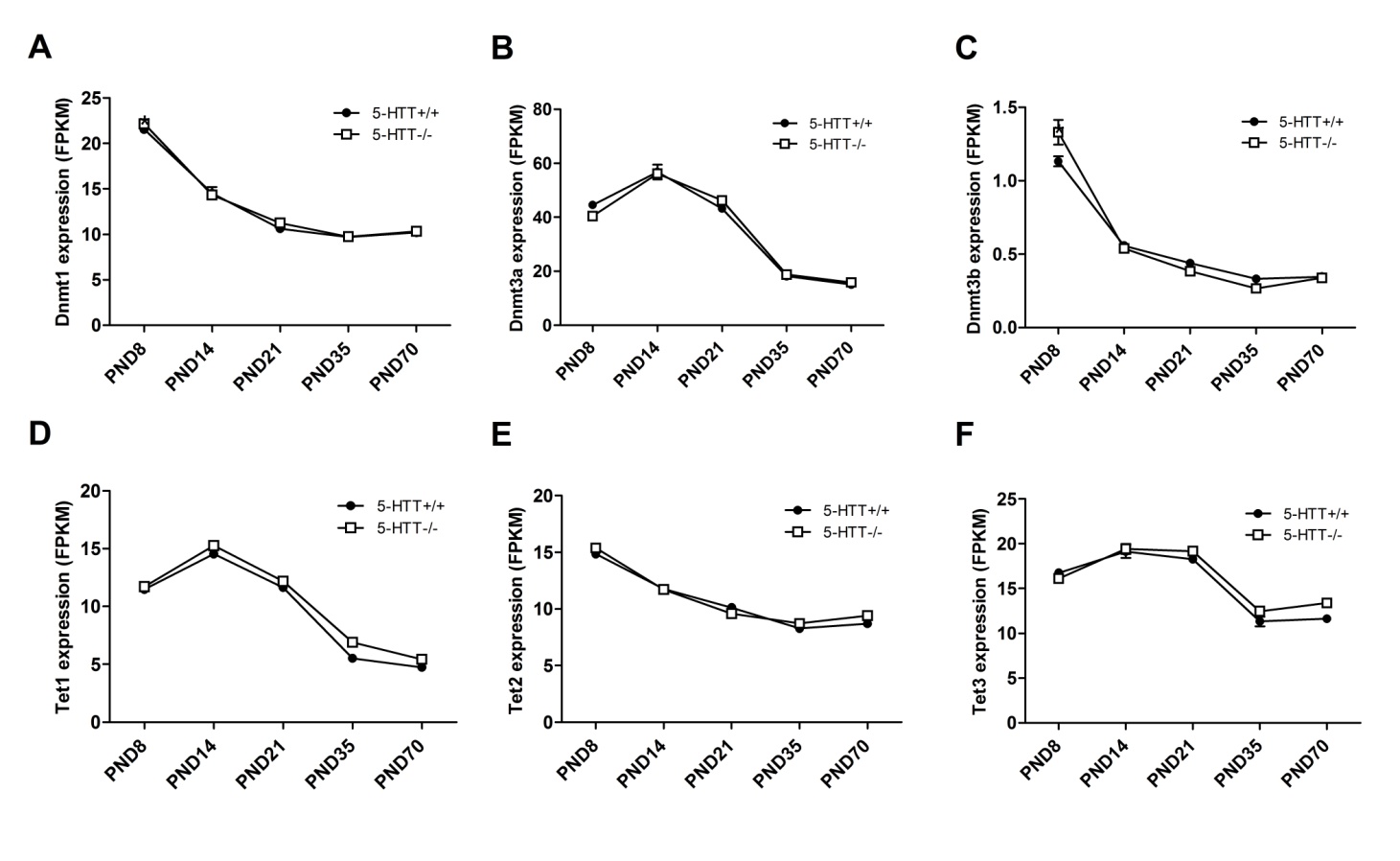
**

**Figure S3: Expression of genes involved in DNA methylation.** A-C: Expression of DNA methyltransferases (Dnmt) across mPFC development. The *Dnmt2* gene is not annotated in the used reference genome (rn4). D-F: Expression of Ten-eleven translocation (Tet) methylcytosine dioxygenases across mPFC development. Y-axis shows expression values in fragments per kilobase per million mapped reads (FPKM). Data are presented as mean +/-S.E.M. of FPKM.

**Figure S4**


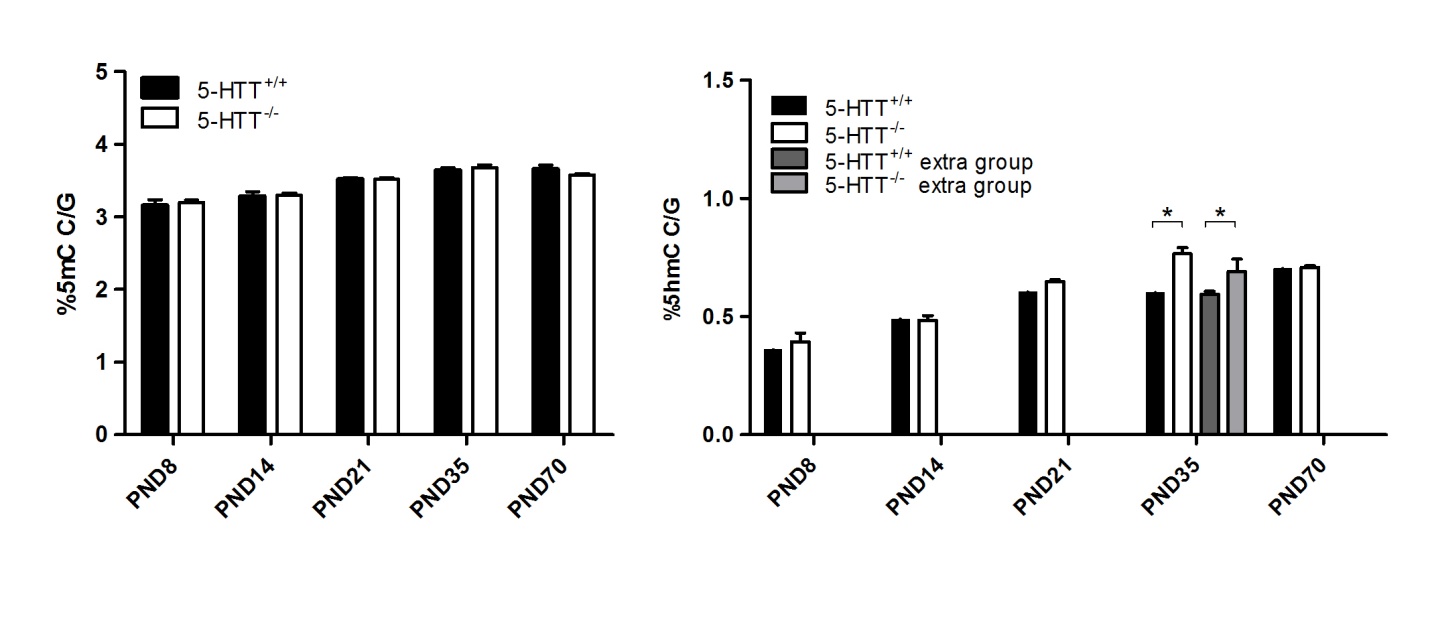


**Figure S4. 5(h)mC measurements in medial prefrontal cortex DNA of 5-HTT^-/-^ and 5-HTT^+/+^ rats.** (A) 5mC levels in 5-HTT^-/-^ and 5-HTT^+/+^ rats measured at five time points (n=5/genotype/time point). 5mC levels were calculated as a concentration percentage ratio of 5-methyl-2’-deoxycytidine/2’-deoxyguanosine (%mdC/dG). (B) 5hmC levels in 5-HTT^-/-^ and 5-HTT^+/+^ rats measured at five time points (n=5/genotype/time point). 5hmC levels were calculated as a concentration percentage ratio of 5-hydroxymethyl-2’-deoxycytidine/2’-deoxyguanosine (%hmdC/dG). 5hmC levels at PND35 were validated using an independent group of rats, depicted as 5-HTT^-/-^ extra and 5-HTT^+/+^ extra (n=10/genotype). *p<0.05.
